# A snapshot of U.S. IRB review of COVID-19 research in the early pandemic

**DOI:** 10.1017/cts.2021.848

**Published:** 2021-09-13

**Authors:** Holly A. Taylor, Kimberley Serpico, Holly Fernandez Lynch, John Baumann, Emily E. Anderson

**Affiliations:** 1 Department of Bioethics, Clinical Center, National Institutes of Health, Bethesda, MD, USA; 2 Office of Regulatory Affairs and Research Compliance, Harvard T.H. Chan School of Public Health, Boston, MA, USA; 3 Department of Medical Ethics and Health Policy, Perelman School of Medicine, University of Pennsylvania, Philadelphia, PA, USA; 4 Office of Research Compliance, Indiana University, Bloomington, IN, USA; 5 Neiswanger Institute for Bioethics, Stritch School of Medicine, Loyola University Chicago, Maywood, IL, USA

**Keywords:** COVID, IRB, turnaround time, review process, content analysis

## Abstract

**Background/Objective:**

Along with the greater research enterprise, Institutional Review Boards (IRBs) had to quickly adapt to the COVID-19 pandemic. IRBs had to review and oversee COVID-related research, while navigating strict public health measures and a workforce largely relegated to working from home. Our objectives were to measure adjustments to standard IRB review processes, IRB turnaround time and document and any novel ethical issues encountered.

**Methods:**

Structured data requests were sent to members of the Consortium to Advance Effective Research Ethics Oversight directing Human Research Protection Programs (HRPP).

**Results:**

Fourteen of the 32 HRPP director members responded to a questionnaire about their approach to review and oversight during COVID-19. Eleven of the 14 provided summary data on COVID-19-specific protocols and six of the 11 provided protocol-related documents for our review. All respondents adopted at least one additional COVID-19-specific step to their usual review process. The average turnaround time for convened and expedited IRB reviews was 15 calendar days. In our review of the documents from 194 COVID-19-specific protocols (*n* = 302 documents), we identified only a single review that raised ethical concerns unique to COVID-19.

**Conclusions:**

Our data provide a snapshot of how HRPPs approached the review of COVID-19-specific protocols at the start of the pandemic in the USA. While not generalizable to all HRPPs, these data indicate that HRPPs can adapt and respond quickly response to a pandemic and likely need little novel expertise in the review and oversight of COVID-19-specific protocols.

## Introduction

Especially in its early days, the COVID-19 pandemic strained the research enterprise in numerous ways. Many studies that were ongoing when the pandemic began had to pause or transition to remote operations and new protocols related to COVID-19 rapidly proliferated. Research institutions, research groups, and Institutional Review Boards (IRBs) faced challenges related to the responsible conduct of research in the face of a public health crisis [1,2,3] and had to quickly adapt to review and oversee COVID-related research, while facing novel policy issues and a workforce largely relegated to working from home. There is limited evidence about how IRBs conduct their work during human and natural disasters [4,5], a gap that has so far persisted with regard to COVID-19. Better understanding these circumstances and IRB responses can help inform future efforts.

Inspired by an analysis of ethics committee reviews of applications for research studies at a single hospital in China during the start of the pandemic there, we sought to characterize the type and volume of COVID-19-specific protocols submitted to IRBs during the first few months of the pandemic in the USA, measure IRB turnaround time on these COVID-19-specific protocols, and describe adjustments to standard IRB review processes in response to COVID-19 [6]. In addition, we reviewed a limited set of IRB materials to explore whether HRPPs encountered any novel ethical and regulatory issues in their review of early COVID-19-specific research protocols.

## Materials and Methods

This project was conducted by a working group of the Consortium to Advance Effective Research Ethics Oversight (www.AEREO.org). AEREO was established in 2018 as a consortium of leaders in human subjects research oversight, research ethics, and empirical methods, with an overall mission to evaluate and improve the effectiveness of IRBs and the overarching Human Research Protection Programs (HRPPs) of which they are often a part. All AEREO members with responsibility for the review and oversight of research at their institution (*n* = 32 at the time of data collection) were invited to participate. AEREO members are affiliated with academic medical centers, higher education institutions, federal agencies, and large health systems.

For the first component of the project, eligible AEREO members were asked to provide information about all COVID-19-specific submissions to their IRB made by May 30, 2020, as well as information regarding the status of those submissions through June 30, 2020. They were instructed to exclude protocols submitted by PIs at their institution collaborating on multi-site research reviewed by a single IRB other than their own. Data collected included study title, type of study and study design (e.g., biomedical, secondary data analysis), type of review (e.g., convened IRB, expedited), and number of days from initial submission to final IRB decision. Respondents were provided with empty tables to allow systematic collection of these data elements. For the second component of the project, respondents were asked to complete a close-ended questionnaire about whether and how they adjusted their review process for COVID-19-specific protocols (e.g., advance administrative review, creation of specialized rapid response IRB panels). Finally, for the third component of the project, respondents were asked to provide key documents (e.g., determination letters, consent forms) for all COVID-19-specific protocols. The project team tallied the number and types of documents provided and created an abstraction form to isolate key information for each protocol. Three members of the study team (HAT, KS, and EEA) reviewed the submitted documents and abstracted the relevant information. The abstraction form for each set of protocols included the key information noted just above (e.g., study title, type of study) and two open-ended questions regarding more substantive issues: 1) protocol issues given scrutiny and 2) ethical issues noted in documents provided. Narrative responses to these open-ended questions were then categorized as either general issues or COVID-19-specific issues by one member of the team (HAT).

## Results

**Fig. 1.**
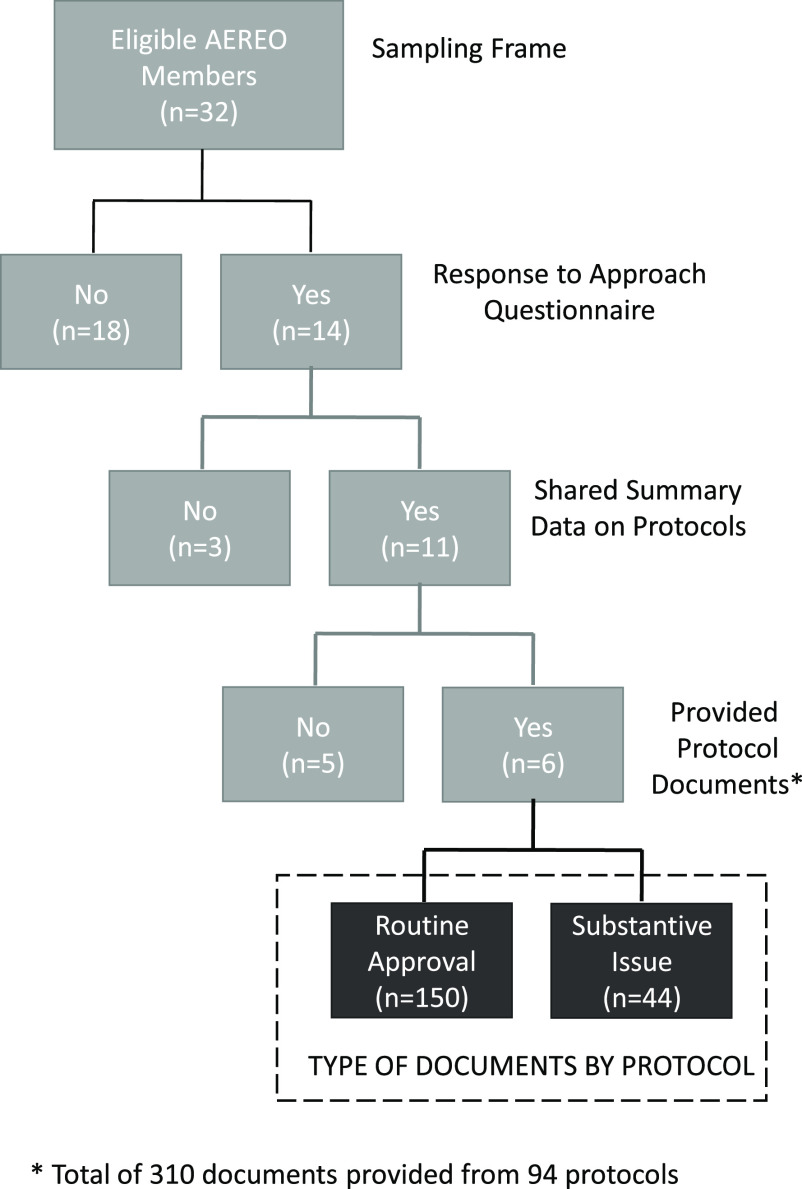
Study flow. AEREO, Consortium to Advance Effective Research Ethics Oversight.

Data submission remained open until November 1, 2020. Fourteen of 32 eligible AEREO members (44%) provided data about their institutions in response to at least one component of the project (Fig. 1). Ten of the fourteen (71%) were from academic medical centers (AMCs), reflecting the broader AEREO membership; of the eighteen members who did not participate, 14 (77%) were AMCs.

In general, all non-COVID in-person research was paused at participating institutions. All 14 institutions responded to the questionnaire about their approach to IRB review for COVID-specific protocols (Fig. 1; Table 1; See Appendix for Data Collection Tools). Nearly three-quarters of HRPPs (10 out of 14) selected the choice indicating that they conducted an administrative review in advance of sending COVID-19-specific protocols to their IRBs, which we understood to mean an *addition*al COVID-19-specific protocol administrative review. Almost two-thirds of institutions prioritized the review of COVID-19-specific protocols ahead of any other protocols (9 out of 14). Almost half of the institutions (6 out of 14) held additional or more frequent IRB meetings to accelerate the pace of review. A number of institutions reported additional activities (*n* = 6) in response to COVID-19, such as providing additional education and hands-on support to investigators, expediting ancillary reviews (e.g., conflict of interest), coordinating biospecimen collection across departments to facilitate COVID-19-specific research, and engaging with individuals from the local community intended to be representative of those likely to be asked to participate in COVID-19 vaccine trials.

**Table 1.** Approaches to review for COVID-specific protocols

	Totals (n = 14), n (%)
Administrative review by HRPP staff in advance of IRB review	10 (71.4)
Adoption of prioritization scheme for review of different types of COVID-19 protocols to prioritize which to be reviewed by the IRB	9 (64.3)
Holding additional or more frequent meetings of existing IRB(s)	6 (42.9)
Activation of *existing* specialized, rapid response IRB	3 (21.4)
Prioritization scheme for protocols recruiting from same sample population by committee other than IRB	3 (21.4)
Creation of *novel* specialized, rapid response IRB	2 (14.3)
Other	6 (42.9)

A single institution may have adopted more than one of these approaches.

HRPP, Human Research Protection Program; IRB, Institutional Review Board.

Eleven institutions provided summary data on COVID-19-specific protocols, type of review, and turnaround time. In total, these 11 institutions reviewed 822 COVID-19-specific protocols over approximately three months from March–May 2020; because each institution reported data only on protocols for which they served as the IRB of record, this number reflects 822 distinct protocols, without overlap between participating sites. Respondents characterized more than one-half of the COVID-19-specific protocols reviewed at their institutions as social science/behavioral studies (*n* = 433) and nearly one quarter as biomedical (*n* = 196). The remainder comprised proposals to conduct secondary data analysis, to create registries, or for compassionate use of treatments not yet approved by the Food and Drug Administration (Table 2). Just over half of the COVID-19-specific protocols were deemed exempt from IRB review, likely corresponding to the number of social/behavioral studies; approximately one-quarter were expedited, and only 5% were sent for review by a convened IRB (Table 3). The average turnaround time was about two weeks for COVID-19-specific protocols subject to convened IRB review (15.8 calendar days), as well as for expedited review (15.4 calendar days), and less than two weeks for exempt COVID-19-specific protocols (10.8 calendar days) (See Table 3).

**Table 2.** Summary of COVID-19 studies reviewed by eleven institutions

Type of study	Total, n (%)
Biomedical	196 (24)
Drug prevention	1
Drug treatment	15
Vaccine	1
Device	15
Genetics/viral sequencing	10
Other (specimen collection)	154
Secondary data analysis	130 (16)
Registry	49 (6)
Data/specimens	48
Other	1
Social/behavioral	433 (53)
Observational	5
Survey	196
Interview	43
Other	189
Expanded access	14 (1)
Total	822

**Table 3.** Review mode and turnaround time for new COVID-19 studies

	Total new initial submissions, n (%)	Average calendar days in review
Convened IRB	41 (5)	15.8
Expedited	181 (24)	15.4
Exempt	421 (55)	10.8
Not HSR	30 (4)	N/A
Other*	75 (10)	N/A
Withdrawn	1 (0.1)	N/A
Pending	14 (2)	N/A
Total	763	N/A

Does not include amendments to existing studies.

* Institutions who differentiated projects not federally regulated or minimal risk.

HSR, Human Subject Research; IRB, Institutional Review Board.

Six institutions provided key documents, totaling 310 documents related to 194 COVID-19-specific protocols (e.g., IRB applications, determination letters, correspondence between IRB and investigator). For each set of documents submitted, we abstracted relevant information about administrative, regulatory, and/or ethical issues identified. Documents submitted for 150 of the COVID-19-specific protocols were straightforward approval letters with institutional administrative boilerplate language. Documents for the remaining 44 protocols included substantive narrative information to explore further. Almost all of these documents (*n* = 42 protocols) included references to “standard” administrative/regulatory deficiencies with the submissions unrelated to COVID-19, such as incomplete study team rosters, missing documentation of study team training, missing study tools/other attachments, study materials in need of translation into other languages, and inconsistencies between study documents. We found that the documents for one-third (*n* = 15) of the 44 COVID-19-specific protocols identified at least one general ethics concern not specific to COVID-19, falling into six categories: risk, study design, privacy, respect for persons, investigator-participant power differential, and benefit, with the latter two categories being least common. One institution adopted COVID-19-specific administrative/regulatory requirements, leading to questions for four protocols about institutional approval to conduct the research, institutional approval to be on site during restricted hours, and compliance with campus COVID-19 prevention measures. This institution also identified COVID-19-related ethical concerns about one protocol, two of which were related to risk and one to study design.

## Discussion

IRBs have faced a number of challenges during the COVID-19 pandemic, the most important of which is the need to rapidly review an influx of applications in a way that will avoid unnecessary delays in promising science during a public health emergency, while simultaneously making sure that the rights, welfare, and interests of research participants are appropriately protected. Our data provide a snapshot of how a group of HRPPs approached the review of COVID-19-specific protocols at the start of the pandemic in the USA, as well as the number and types of studies reviewed during this time and IRB turnaround speed. Our review of COVID-19-specific protocols determined that IRBs were identifying the same type of administrative, regulatory, and ethical concerns in these protocols as those typically identified in the general review of research. Common approaches at institutions in our sample included efforts to manage IRB workloads (and other institutional resources) through administrative review prior to board review and efforts to increase IRB responsiveness by moving COVID-19-specific protocols to the front of the queue, holding additional meetings, or relying on COVID-19-specific review panels. IRBs received a substantial number of new protocols but were able to take advantage of regulatory pathways intended to minimize burden, including exemption and expedited review, for the vast majority of them. This likely enabled IRBs to focus their attention on the smaller number of COVID-19-specific protocols in need of more significant oversight due to increased risk and complexity.

One common complaint about IRBs is that they are a source of administrative delay in the research enterprise [7]. As a result, researchers, boards, and institutions have traditionally paid close attention to turnaround time – broadly understood as the number of calendar days from research application submission to approval by the IRB [8]. However, turnaround time can be affected by multiple factors, not all of which are within the IRB’s control. Some ancillary reviews, such as conflict of interest or radiation safety for example, may impact the speed of making a final determination as to whether research can proceed. Study teams also may be slow to respond to IRB stipulated changes to protocols.

Newsom and colleagues conducted a survey of fifty-five IRB Directors at institutions accredited by the Association for the Accreditation of Human Research Protection Programs, Inc. (AAHRPP) [9]. They asked IRBs to report the number of calendar days from “submission of a full board study to review to approval,” finding mean time of 40.3 calendar days. They asked similar questions about expedited and exempt reviews: “days from submission of an expedited study to approval,” and “days from submission of an exempt study to determination” and found mean times of 20.1 and 11.7 calendar days respectively. By comparison, the IRBs in our sample completed their full board reviews of COVID-19-specific protocols 2 and a half times faster, expedited reviews 5 days faster, and exempt reviews in about the same amount of time.

In a more recent single-site study testing whether different levels of effort by a regulatory service group (low v. high) reduced turnaround time from submission to approval the average turnaround time for full board review for proposals that received high effort was 68.7 calendar days and 52.6 calendar days for expedited reviews [10]. In a similar single-site study, Sonne and colleagues offered extra regulatory services help study teams with their submissions. The mean turnaround time for an expedited review was 73.4 calendar days for the study teams who sought help compared to 178.6 calendar days for the study teams that did not seek help [11]. The turnaround times among the IRBs in our sample were quick by comparison Importantly, rapid turnaround time could reflect taking insufficient time to review a protocol, whereas slow turnaround could simply reflect complexity of the issues relevant to the IRB’s determination. The goal is not necessarily speed but rather efficiency, that is, taking the time needed to make good decisions that adequately address ethical issues and comply with the regulations, but not any more time than that.

Although we are not able to make a judgment about the efficiency of protocol reviews in our sample, on average, they were relatively rapid. Time is of the essence during a pandemic, but our data do not suggest lengthy delays at the IRB stage. To the contrary, they suggest that participating institutions were able to rapidly respond to heightened protocol volume, while turning protocols around in a timely fashion.

Measuring the substantive quality of IRB review is a perpetual challenge, making it difficult to assess whether the pandemic had any impact on review quality [12]. In our small sub-sample, IRBs were clearly reviewing protocols thoroughly enough to identify routine administrative and regulatory deficiencies, as well as raising ethical concerns in some cases. This finding suggests that, even during extreme circumstances, IRBs are following standard operating processes that systematize review in consistent and reliable ways. However, we did not seek to compare our findings against pre-pandemic protocols, for example, or to evaluate the overall quality of reviews.

## Limitations

The main limitation of this study is that it is a convenience sample comprised of AEREO members rather than a broader national survey of registered IRBs or a national sample of academic medical centers. AEREO members are a self-selected group composed of leaders in ethics and human subject protections. Thus their preparation, procedures, and practices might be different – and likely stronger – than an unselected group of AMCs and other health research institutions, potentially limiting the generalizability of these findings. In addition, some data are missing due to the fact that HRPPs/IRBs track different outcomes. We received requested documents from only a small number of participating institutions due to concerns about confidentiality and the time needed to collate materials when HRPPs were already facing heightened pandemic obligations. Finally, we only collected data from the first three months of the pandemic. All of the above limited our ability to draw generalizable conclusions about ethical and regulatory challenges raised in COVID protocols in general.

## Conclusion

IRBs are a critical component of the research enterprise and must be able to mobilize in response to a public health emergency. Our data suggest that the institutions in our sample were able to do so successfully, including through the adoption of a number of approaches that may also be useful in non-pandemic circumstances and for other high-priority research. For example, IRBs should consider having plans in place to accelerate their pace of review and to prioritize among projects addressing the public health threat at hand and/or among projects planning to collect data from the same sample populations [3]. Yet there are several unanswered questions. Future work could build on our preliminary assessment by further examining the details of how institutions reviewed COVID protocols, seeking to understand stakeholder perspectives on the challenges and quality of review under pandemic conditions (for example through interviews with HRPP directors and researchers), and considering the resources needed to support IRBs when flexibility is paramount. Overall, it is also important to continue efforts to better understand what IRB quality means and how it should be measured so that we can avoid, identify, and address quality concerns when IRBs face new stressors.

## Supplementary material

To view supplementary material for this article, please visit https://doi.org/10.1017/cts.2021.848.
