# Supplementary material for: A snapshot of U.S. IRB review of COVID-19 research in the early pandemic
Source: J Clin Transl Sci. 2021 Sep 13;5(1):e205. doi: 10.1017/cts.2021.848 (PMC8692853; doi:10.1017/cts.2021.848)
Supplement: Supplementary file 1 [file S2059866121008487sup001.pdf]

AEREO COVID-19 IRB PROJECT

How is your site approaching the review of COVID-19 protocols? (Check all that apply)

- ❐ Administrative review in advance of IRB review
- ❐ Scientific review in advance of IRB review
- ❐ Holding additional or more frequent meetings of existing IRB(s)
- ❐ Activation of specialized, rapid response IRB
- ❐ Creation of specialized, rapid response IRB
- ❐ Adoption of prioritization scheme for review of different types of COVID-19 protocols (in advance of IRB review)
- ❐ Prioritization scheme for protocols recruiting from same sample population by IRB
- ❐ Prioritization scheme for protocols recruiting from same sample population by committee other than IRB
- ❐ Other, please specify

  ____

  ____

  ____

  ____
